# Spontaneous Regression (SR) of Male Breast Cancer (MBC): A Rare Case Highlighting Diagnostic Pitfalls and Management Caution

**DOI:** 10.1155/cris/1816717

**Published:** 2025-10-03

**Authors:** Jia Chyi Tay, Woon Teen Sia, Nadzrin Md Yusof, Li Jie Thee

**Affiliations:** ^1^Department of Surgery, Hospital Sultanah Bahiyah, Alor Setar, Kedah, Malaysia; ^2^Department of Surgery, National University of Malaysia, Bangi, Selangor, Malaysia; ^3^Monash Medical University, Subang Jaya, Selangor, Malaysia; ^4^Department of Pathology, Hospital Sultanah Bahiyah, Alor Setar, Kedah, Malaysia

## Abstract

**Background:**

Male breast cancer (MBC) is rare, accounting for only 1.8% of all breast cancer cases diagnosed globally. However, there is a rising trend in its incidence over the past decades. Spontaneous regression (SR) of a tumor, on the other hand, is a rare but well-documented phenomenon.

**Case Presentation:**

We reported a case of MBC that showed SR in the surgical specimen after the histopathologic diagnosis of invasive breast cancer in the core needle biopsy sample. A 58-year-old gentleman presented with a palpable left retroareolar mass, nipple retraction and intermittent pain for 2 months. Imaging and histopathological examination (HPE) confirmed an estrogen- and progesterone-receptor-positive, HER-2 negative invasive carcinoma, which was treated with left mastectomy with axillary clearance (MAC). Intraoperatively, no breast mass was palpable, and SR of the tumor was reported for the surgical sample.

**Conclusion:**

This case not only emphasizes the rarity of MBC but also draws attention to the exceptional phenomenon of SR in invasive carcinoma. Recognition of such rare events underscores the importance of cautious decision-making, multidisciplinary management, and further research into the biological and immunological mechanisms underlying tumor regression.

**Summary**

• Spontaneous regression (SR) in male breast cancer (MBC)is an exceptionally rare phenomenon.
• This case underscores the critical role of histopathological confirmation and multidisciplinary evaluation in guiding management, highlighting the necessity for heightened clinical awareness to navigate diagnostic uncertainty and prevent unnecessary therapeutic interventions.

## 1. Introduction

Male breast cancer (MBC) is rare, accounting for only 1.8% of all breast cancer cases diagnosed globally [1, 2]. However, over the past decade, its incidence has shown a rising trend, which is concerning due to limited research and treatment guidelines. Recent literature reveals that aging, a positive family history, specific gene mutations, hormonal imbalances, and unregulated expression of hormone receptors are significant risk factors for MBC [1, 3]. A painless palpable subareolar lump is the most common symptom in MBC [4]. Other symptoms such as nipple retraction, nipple ulceration ± bleeding, axillary lymphadenopathy, skin erythema, and flakiness are similar to the female breast cancer (FBC) [1, 5]. MBC tends to be the ductal type and receptor positive, with poorer overall survival than females [6].

Spontaneous regression (SR) of cancer is a rare but well-documented phenomenon which was first mentioned in the Ebers Papyrus of 1550 BCE [7]. It is defined as partial or complete remission of primary or metastatic tumor tissues in the absence of treatment [8]. The incidence of SR was reported to be approximately 1 in every 60,000–100,000 malignancies [9]. Although SR has been reported in breast cancer, the information and understanding remain scarce. Current literature regards the roles of the host's immunological responses and oncotic apoptosis as the major drives of its pathogenesis [8–10].

This case report highlights the challenges of diagnosing breast cancer in a male patient and brings awareness to the SR in invasive breast cancer.

## 2. Case Presentation

A 58-year-old gentleman presented to the surgical clinic for a palpable mass in the left retroareolar region, associated with nipple retraction and on-and-off pain for 2 months. He denied nipple discharge and had no history of trauma to the breast. He experienced loss of appetite for 2 weeks but no significant loss of weight. His medical history was significant for ischemic heart disease, dyslipidemia, and type 2 diabetes mellitus. These conditions were managed conservatively with oral medications (isosorbide dinitrate, aspirin, simvastatin, and metformin) and were well-controlled. He was a nonsmoker and nondrinker. There was a positive family history of colorectal cancer in his family.

Clinically, he was noncachectic, with all vitals within normal limits. On physical examination, a 3 cm × 4 cm firm mass was palpable at the left retroareolar region, slightly mobile, nontender, and not tethered to skin. The skin appeared normal, with no scar, scaling, erythema, and peau d'orange seen. The nipple was retracted, but there was no discharge on compression.

A bilateral breast ultrasound examination and Trucut biopsy were performed. Ultrasound showed a well-defined heterogenous hypoechoic solid lesion at left retroareolar region, measuring 1.4 cm × 1.8 cm × 1.4 cm (Anteroposterior [AP] × Width [W] × Craniocaudal [CC]). No intralesional vascularity was demonstrated, but malignancy could not be excluded. The Trucut biopsy showed an equivocal result. An ultrasound-guided biopsy was then performed, and the histopathological examination (HPE) showed an estrogen- and progesterone-receptor-positive, HER-2 negative invasive carcinoma of the breast, at least Grade 2.

A contrast-enhanced computed tomography (CECT) thorax/abdomen/pelvic was done subsequently for staging. The left breast lesion (2.2 cm AP × 1.7 cm W × 1.8 cm CC) was consistent with primary malignancy, no significant mediastinal or hilar lymphadenopathy, no focal liver and renal lesion, no significant abdominopelvic lymphadenopathy, and no suspicious bone lesion. However, there was a lung nodule with an irregular margin seen at the posteromedial segment of the left lower lobe, measuring 1.0 cm x 2.0 cm (AP × W), and homogenous enlargement of prostate, measuring 3.4 cm × 4.1 cm × 4.7 cm (AP × W × CC) which warranted further investigations.

A positron emission tomography–computed tomography (PET-CT) with tracer (F-18 Fluorodeoxyglucose [FDG]) was subsequently performed, accompanied by a contemporaneous low-dose unenhanced CT for attenuation correction and anatomical localization. FDG avidity was seen in the left breast lesion. The lung focus appeared hypermetabolic and was initially suggestive of metastasis, while the prostate lesion was FDG-avid and required further evaluation to exclude malignancy.

A multidisciplinary team (MDT) meeting involving oncologists, radiologists, surgeons, and pathologists was organized for discussions and decision-making of the treatment plan. Careful comparison between the earlier diagnostic contrast-enhanced CT and the subsequent low-dose CT performed during PET-CT showed that the lung nodule previously noted was no longer visualized, supporting the impression of transient infective changes rather than metastasis. Given the aggressive nature of MBC, the MDT recommended proceeding with a left mastectomy and axillary clearance (MAC) as the primary treatment, while referring the patient to urology for further assessment of the prostate lesion.

The surgery was performed approximately 3 months after the MDT decision, following further evaluation of the prostate lesion, the patient's decision-making process, and scheduling considerations. Neoadjuvant therapy was discussed but deferred, as the MDT favored upfront surgery in view of the ER/PR-positive, HER-2-negative biology, and absence of confirmed metastasis. A left MAC was performed. Intraoperatively, no palpable breast mass was found, but multiple enlarged axillary nodes were noted and excised. The breast specimen with 25 lymph nodes were submitted for histopathological and microscopic examination.

**Figure 1:**
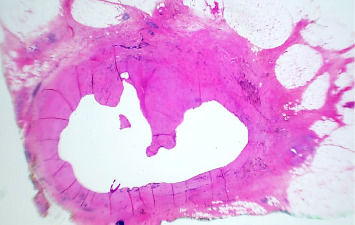
Low-power view showing the only suspicious fibrotic area with a cystic lesion (H&E, 40x).

**Figure 2:**
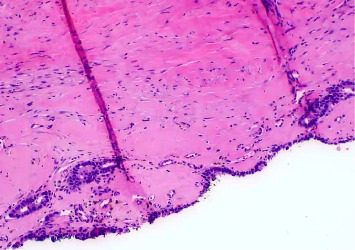
High-power view of the cystic lining epithelium, demonstrating a two-layered architecture with intact myoepithelial cells and minimal inflammatory infiltrates (H&E, 200x).

**Figure 3:**
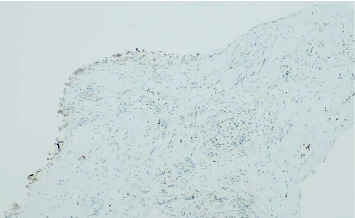
P63 immunostaining confirming the presence of myoepithelial cells, ruling out invasive carcinoma (IHC, 200x).

Microscopic examination revealed no residual invasive carcinoma. One cystic lesion was identified, lined by minimally intact two-tiered epithelial and myoepithelial cells, confirmed by P63 immunostaining. (Figures 1, 2, and 3). Focal aggregates of hemosiderin-laden macrophages were noted along the cyst wall, suggestive of prior hemorrhagic or regressive changes. The surrounding stroma showed fibrotic hyalinization with a few benign glands and scattered chronic inflammatory infiltrates. The remaining breast tissue demonstrated benign terminal duct–lobular units, with no evidence of ductal carcinoma in situ or malignancy across all sampled sections. Pathologist reported SR of the tumor as all margins of the specimen showed no evidence of carcinoma in situ or invasive malignancy.

The patient recovered well from surgery and was commenced on tamoxifen, 20 mg once daily. Although no residual carcinoma was identified in the surgical specimen, adjuvant endocrine therapy was initiated given the biopsy-proven ER/PR-positive invasive carcinoma and the potential risk of micrometastatic disease and recurrence. He was followed-up every 3 months with a breast ultrasound in the surgical clinic, while management of his prostatomegaly was taken over by the urology team.

## 3. Discussion

SR of cancer is a rare but well-documented phenomenon in the literature which its pathogenesis and clinical significance are not fully understood [11, 12]. Almost all histological types of malignant neoplasm could regress spontaneously despite the differences in frequencies [10]. SR is typically regarded as a result of the activation of apoptosis by immunological responses, tumor microenvironment, and DNA oncogenic suppression [8, 10]. It is hypothesized that an efficacious immune system suppresses the growth and proliferation of cancer cells in the tumor microenvironment, resulting in a relatively higher number of natural killer cells than circulating cancer cells in the blood, leading to SR. With that, SR is particularly associated with the presence of inhibitors of metalloproteinases, angiogenesis, and scarcity of specific proteins in the tumor microenvironment [10].

SR of breast cancer is rarer than other types of malignancies, such as testicular germ cell tumors, melanoma, cutaneous basal cell cancers, and renal cell cancer [8]. The number of reported cases significantly decreased in recent years since the administration of effective treatments. The activation of apoptosis in breast cancer involves T-cell-mediated mechanism (i.e., Fas–Fas Ligand, TNFa–TNFa receptor, perforin-granzyme pathway) and p53 gene activation [13]. Significantly, p53 gene activation is active in almost half of the breast cancer cases and exceptionally active in hormone-receptor-positive cases [14, 15].

With <1% of incidence, MBC is uncommon worldwide and not well publicized, especially in the Asian populations [16]. Based on a local retrospective study published in 2009, Malaysian men were at risk of breast cancer, although the prognosis was better compared to that of female patients [16]. MBC is more likely to present at an advanced stage due to stigmata in the health-seeking behavior and delayed diagnosis [16, 17]. The vigilance of clinicians in diagnosing male breast lumps is crucial to avoid misdiagnosis. The predisposing risk factors of MBC include radiation exposure, estrogen use, hypoestrogenism (e.g., liver cirrhosis, Klinefelter's syndrome), positive family history of breast carcinoma, gene inheritance, and mutations (e.g., BRCA1, BRCA2, and TP53 mutations) [17]. The majority of the patients present with a retroareolar mass, which is consistent with our case. Triple assessment has been widely adopted to diagnose MBC [18].

According to the America College of Radiology, breast imaging (e.g., mammography and ultrasonography) should be performed when MBC is suspected, while any suspicious findings should be confirmed with a core biopsy. Histopathologic classification of tumor types is necessary to guide treatment [19, 20]. A MAC remains as the primary treatment of MBCA, while tamoxifen is often recommended as the systemic therapy [21, 22]. The American Society of Clinical Oncology (ASCO) recommends that men with early-stage hormone receptor-positive (HR+) breast cancer receive adjuvant tamoxifen for 5 years. For patients who tolerate therapy well and remain at high risk of recurrence at 5 years, continuation of treatment for an additional 5 years is advised [23].

A MDT approach involving radiologists, oncologists, surgeons, and pathologists is essential for the diagnosis and management of MBC due to its unique challenges. MDT discussions would effectively improve the accuracy in radiologic–pathologic correlation, provide personalized and evidence-based treatment decisions, resulting in higher 5-year survival rates [24, 25]. Neoadjuvant therapy is sometimes considered in MBC, particularly in cases with large tumors, locally advanced disease, or confirmed distant metastases [26]. In our patient, this option was discussed at the MDT meeting. However, the tumor was biopsy-proven estrogen and progesterone receptor-positive and HER-2 negative, with no evidence of confirmed metastatic spread after repeat imaging and MDT review. Moreover, the patient required optimization of ischemic heart disease and diabetes before any systemic therapy or surgery. Given these considerations, the MDT concluded that upfront surgery would provide both diagnostic and therapeutic benefit, while avoiding potential delays or risks of systemic treatment in a comorbid patient. This decision underscores the importance of individualized management in MBC and highlights the central role of MDT evaluation in guiding safe and effective treatment.

## 4. Conclusion

In addition to the rare entity of MBC, our case brought attention to the SR of breast cancer. Diagnosis of MBC was challenging, and a multidisciplinary approach was recommended. In particular, the team opted for upfront surgery rather than neoadjuvant therapy, given the tumor's HR+, HER-2 negative biology, and the absence of confirmed metastasis. Biopsy and HPE were pivotal in guiding management and ultimately revealing SR. This case underscores the importance of vigilance in evaluating male breast lumps, exercising caution in surgical decision-making, and recognizing rare regression phenomena. Moreover, the observation of SR opens new perspectives for research into the immunological environment and tumor–host interactions, providing valuable insights into cancer biology and novel therapeutic strategies.

## Data Availability

The research data are not shared.
